# Management of Tuberculous Cutaneous Fistula

**DOI:** 10.1155/2020/7840963

**Published:** 2020-02-10

**Authors:** Massamba Miabaou Didace, Lenga Loumingou Ida, Ondima Irène, Peko Jean Félix

**Affiliations:** ^1^Department of Digestive Surgery, Brazzaville Teaching University Hospital, Brazzaville, Congo; ^2^Department of Dermatology, Brazzaville Teaching University Hospital, Brazzaville, Congo; ^3^Department of Pediatrics Surgery, Brazzaville Teaching University Hospital, Brazzaville, Congo; ^4^Laboratory of Pathological Anatomy and Cytology, Brazzaville Teaching University Hospital, Brazzaville, Congo

## Abstract

Tuberculosis is an endemic emergency that is prevalent in developing countries, particularly in sub-Saharan Black Africa, including Congo-Brazzaville. In addition to the pulmonary, ganglionic, and bone forms, there are other poorly documented locations. In the Congo, among these is cutaneous tuberculosis which is exceptional. A 9-year-old boy and two adult patients had persistent lesions of the left hip and thigh wounds, chest wall, and hypogastric wound with no healing for more than four months, respectively. Among these patients, one case of tuberculous contact was noted. Histopathological examination revealed a Koester follicle, suggesting a tuberculous skin fistula. A fistulectomy was performed, coupled with a quadruple antituberculous therapy combining rifampicin, isoniazid, ethambutol, and pyrazinamide for two months, relayed by a dual therapy consisting of isoniazid and ethambutol for 6 to 8 months. The evolution was favorable in all cases with healing of the lesions after 3 to 6 weeks. The existence of inexhaustible fistulas and the absence of scarring of a wound should make one suspect, among other things, cutaneous tuberculosis. The product of fistulectomy makes it possible to establish the histological diagnosis of cutaneous tuberculosis.

## 1. Introduction

Tuberculosis is a ubiquitous affliction with multiple facets. The pulmonary form is by far the most frequent [1]. Although the total prevalence of tuberculosis on a world level is declining, it remains nevertheless 250 times higher in sub-Saharan Black Africa than in the developed countries [2]. Tuberculosis remains a public health problem in Africa. According to projections of the World Health Organization, 9.7 million people will have contracted tuberculosis in 2020 [3]. Except for the pulmonary forms of other extrapulmonary localizations (15 to 20% of all the cases of tuberculosis), Congo-Brazzaville saw their number increased with the advent of coinfection by HIV AIDS, the appearance of multiresistant stocks, the migrations of the populations following wars, and the number of growing people in precarious social conditions. Among the rare forms, the cutaneous tuberculosis must be present in memory. It evolves spontaneously into an ulceration, with the formation of an abscess or wounds leaving the flow of the friable pus without any tendency for cicatrization. Faced with the poor response to anti-tuberculosis treatment, the Infectious Disease Society of America (IDSA) recommends surgical excision in certain circumstances [4], as mentioned in several studies [5–7]. In Congo, no study has been performed on surgical excision in patients with tuberculous cutaneous fistula. We report three observations in which it posed a real diagnostic problem because all the patients were received at the stage of fistulation.

The interest of this preliminary work lies in the fact that it illustrates the problems of the assumption of responsibility of the cold abscesses and the cutaneous fistulations oozing or productive in the African black medium.

## 2. Case Presentation

### 2.1. Case Report 1

**Figure 1:**
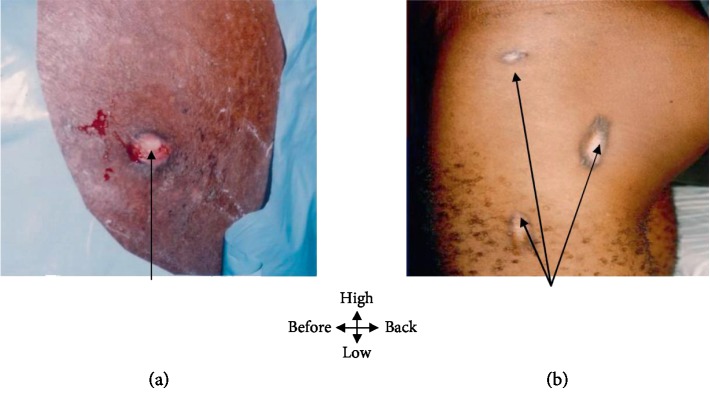
(a) Hip fistula and left thigh; whitish pus with creamy debris. (b) Fistula healing at 30 days.

A 28-year-old patient is admitted to a dermatology clinic for lack of healing of left hip and thigh wounds that have been evolving for 16 months. He had a general condition on the admission examination which was slightly altered (fever at 38°C and an estimated weight loss of 11%), with macroscopic lesions showing fistulas of the left hip and thigh with discharge of pus whitish with creamy debris (Figure 1). The biological explorations reveal a moderate inflammatory anemia, an accelerated VS (145/155). The search for acid-fast bacilli in secretions was negative. Retroviral serology was positive. Histological examination of the fistulectomy product identified Koester follicles. Radiography of the left hip and thigh was normal. The combination of these epidemiological and paraclinical data suggested the diagnosis of tuberculous cutaneous fistula. The treatment consisted of fistulectomy under locoregional anesthesia (rachianesthesia), complemented by an antituberculous quadruple therapy combining rifampicin, isoniazid, ethambutol, and pyrazinamide for 2 months then a relay made of isoniazid and ethambutol for 4 months. Treatment progress was marked by scarring after 30 days (Figure 1).

### 2.2. Case Report 2

**Figure 2:**
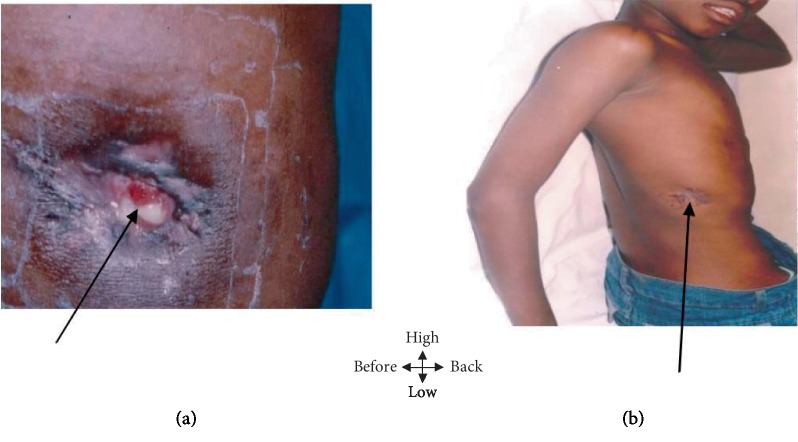
(a) Fistula of the base of the hemithorax straight with purplish irregular edges letting off whitish pus with creamy debris. (b) Fistula healing at 21 days.

A 9-year-old child was admitted to pediatric surgery for a wound in the wall of the right hemithorax without any tendency to scar for 6 months. No notion of tuberculous contagion was found. On examination, a weight loss of 5% was noted, as was a cutaneous fistula of the base of the right hemithorax with irregular and purplish margins, leaving whitish pus with creamy debris (Figure 2). Paraclinical investigations revealed an acceleration of the sedimentation rate (25/50), serology of HIV was negative, and erosion of the 9th and 10th straight ribs, as well as the identification of a Koester follicle on histological examination. This clinical and paraclinical assessment led to the diagnosis of tuberculous cutaneous fistula. The treatment consisted of fistulectomy associated with bone curettage and quadruple antituberculous therapy for 8 months (rifampicin, isoniazid, ethambutol, and pyrazinamide for 2 months, followed by isoniasis relapse and ethambutol for 6 months). Healing of the wound was obtained after 21 days of treatment (Figure 2).

### 2.3. Case Report 3

**Figure 3:**
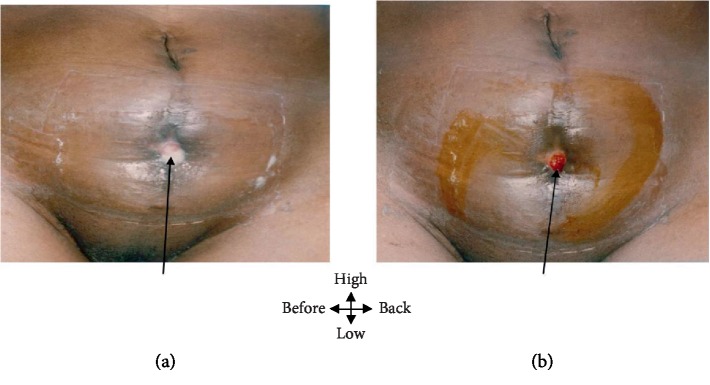
(a) Pelvic fistula whitish pus with creamy. (b) 21-day healing fistula.

A 57-year-old patient admitted to visceral surgery for a lack of healing of a wound of the hypogastric wall that has been evolving for 8 months, in the aftermath of drainage of an abscess of the pelvic wall. There is an antecedent of tuberculous contagion. On examination, the general condition is slightly altered with a fever at 37°C and a weight loss of 8%. There is also a pelvic fistula with indurated edges, letting off whitish pus with creamy debris (Figure 3). Biological exploration revealed moderate inflammatory anemia and acceleration of sedimentation rate (98/110); serology of HIV was negative. The pieces of biopsy have objectified a follicle of Koester. Ultimately, the diagnosis of tuberculous cutaneous fistula was made. The treatment was identical to that of the second patient (observation 2). Healing of the wound was achieved after 44 days of TB treatment (Figure 3).

## 3. Discussion

The cutaneous attack of tuberculosis is not very frequent. Indeed, it represents less than 1% in the Occident [8]. In countries in the process of development like ours, one has to expect an increase in the forgotten forms of this disease with the recrudescence of the pulmonary form related to the syndrome of the acquired immunodeficiency, coupled with the low socioeconomic level as in our observations.

Cutaneous tuberculosis accounts for 2 to 3% of the whole of the attacks of the disease. Its prevalence is not known in our country. In Madrid, Farina et al. [9] report 11 cases in 14 years. In South Africa, Visser and Heyl [10] note 92 observations in 10 years. However, this prevalence is variable according to the localization of the dents. For example, Abid et al. [11] report 3 cases in a Moroccan child of 8 years, wherein cutaneous dents reached the thoracic wall of tubercular patients; Peko et al. [12] in the Congo note 5 cases in 7 years. In the same direction, a case of sinus opening cutaneous of the palmar face of the wrist is observed by Monchal et al. [13] in a farmer of the Ivory Coast.

The pathogenic agent of human tuberculosis is the bacillus of Koch. The ways of contamination are generally aerodigestive before gaining cutaneous fabric by lymphatic or hematogenic way starting from the canker of inoculation [14]. The transmission of the bacillus of Koch is primarily a human pathogen, but it can infect and afflict nonhuman primates and other diverse mammals including elephants and dogs. Back-transmission from animals to humans has been suspected in some instances. Also, it must be kept in mind that *M. bovis* tuberculosis generally is grouped with *M. tuberculosis* for purposes of diagnosis and treatment, and the species affinity is different. However, in our work, the concept of the tuberculosis story is still not found.

The role of surgery in the treatment of cutaneous tuberculous fistulae is unresolved. The clearest contribution is diagnosis via biopsy and specialized histopathological methods, including extraction of mycobacterial DNA. When the diagnosis has been made solely from direct physical examination, with foreknowledge of the likelihood of tuberculosis, the standard WHO-recommended treatment regimen for pulmonary tuberculosis probably will be successful for almost all patients who have one of these conditions. Therapeutic surgery would have to be elected case-by-case, in accordance with the likely benefit, perhaps on the basis of structural problems. Two of our three patients presented tuberculous cutaneous fistula at the level of the thoracic and abdominal walls. For the chest wall, bone lesions are frequent [14, 15]; in our case, it was a costal injury. In the literature, the cutaneous dent tubercular patient is observed in the two sexes and at any age but generally in teenagers and young adults. In our study, it was found in a 9-year-old boy. The localization of the intercostal is rare and is well documented in the literature. This form is found when one meets certain factors of risk, such as the difficulties of etiologic diagnosis and the long duration of suitable management of a former antibiotic therapy not specified. However, the pathogenesis of the tubercular patient of the thoracic wall is multifactorial. However, it is allowed that the large medullary veins of activity and the sternum provide suitable conditions for the local development of the acid-proof bacilli; however, the mechanisms of the contamination are still being discussed: prolongation by contiguity of the pulmonary or pleural disease, hematogenic diffusion, and direct inoculation by the transcutaneous prolongation of the lymphadenitis of the wall of trunk [16, 17]. With regard to patient 3 who introduced a wound hypogastric and was 57 years old, this form is found more in children or teenagers and is associated an active or old tuberculosis. In our case, the duration of the disease exceeded 13 months. It was a cold tuberculous abscess fistulated on the skin. Historically, the tuberculous cold abscesses appear like firm subcutaneous nodules, mobile at the beginning; it is the crudeness stage. Then, the nodules softened to form painless fluctuating abscesses; it is the stage of softening. Then, the nodules softened to form painless abscess fluctuations; this is the softening stage. The skin is perforated with the formation of irregular ulcers surrounded by a crust; this is the stage of ulceration.

In Hong Kong, Chong and Lo [18] found various forms of cutaneous tuberculosis. In an Indian study carried out in 1987, Jerajani et al. [19] reported more cases of tuberculous cutaneous fistula. In the three observations presented, the fistula was the consequence of a cold abscess which was incised or fistulated spontaneously.

At first glance, fistulas may suggest either a yeast infection, a gummy syphilis, or a tumor process. But the characteristic of the creamy pus with tissue remains running out through these dents can already direct the clinician towards a tuberculosis.

The bacteriological examination only of the pus can mislead the diagnosis when it highlights germs of superinfection and not the bacillus of Koch itself. In our work, the direct examination with culture on medium of Lohenstein for the identification of the bacillus of Koch appeared negative in the three cases.

The histological study of the biopsies is the examination of choice because it makes it possible in its sinus form to find the elementary lesions of the follicle of Koester on the paraffin cuts colored with the hematein. The bacillus of Koch appears as small red sticks with the Ziehl Neelsen coloring. This confirms the diagnosis of tuberculosis.

In the mycosis, coloring with Grocott-Gomori would highlight yeasts or filaments. In both other cases, there would not be necrosis cases and thus no oozing fistulation. In addition, the lesions would be nodular in sarcoidosis and periannexiellis in tuberculosis leprosy.

The treatment of cutaneous tuberculosis is identical to that of pulmonary tuberculosis output with a longer duration for the latter. Therapy is based on the combination of four antituberculosis drugs associating rifampicin, isoniazid, ethambutol, and pyrazinamide during 6 to 8 months.

Two of the three patients had a surgical drainage of the cold abscess. In theory, one never should incise a tuberculous cold abscess because its wall does not have any tendency to depress; it would persist as a cavity which, after incision, would result in tuberculosis of the skin.

Thus, it is desirable to carry out a block excision of all the encysted abscesses. When debridement of the dent is quietly carried out, and the antituberculous treatment is good, the results are satisfactory with the drying up of the seepage followed by the cicatrization of the wound.

## 4. Conclusion

The existence of inexhaustible dents and the absence of cicatrization of a wound must make one suspect, amongst other things, a cutaneous tuberculosis. In an African black medium, the incidence of the syndrome of acquired immunodeficiency undoubtedly will involve the multiplication of the cases. Any trailing and oozing wound must consequently be the subject of a bacteriological and especially histological examination. However, this last makes it possible to confirm with certainty the diagnosis of cutaneous tuberculosis, responsible for the absence of cicatrization of the wounds; only a specific treatment will allow the draining of the lesions.
